# Reduced Structural Connectivity between Sensorimotor and Language Areas in Rolandic Epilepsy

**DOI:** 10.1371/journal.pone.0083568

**Published:** 2013-12-23

**Authors:** René M. H. Besseling, Jacobus F. A. Jansen, Geke M. Overvliet, Sylvie J. M. van der Kruijs, Saskia C. M. Ebus, Anton de Louw, Paul A. M. Hofman, Johannes S. H. Vles, Albert P. Aldenkamp, Walter H. Backes

**Affiliations:** 1 Epilepsy Center Kempenhaeghe, Heeze, the Netherlands; 2 Research School for Mental Health and Neuroscience, Maastricht University, Maastricht, the Netherlands; 3 Department of Radiology, Maastricht University Medical Center, Maastricht, the Netherlands; 4 Department of Neurology, Maastricht University Medical Center, Maastricht, the Netherlands; University Children's Hospital, Germany

## Abstract

**Introduction:**

Rolandic epilepsy (RE) is a childhood epilepsy with centrotemporal (rolandic) spikes, that is increasingly associated with language impairment. In this study, we tested for a white matter (connectivity) correlate, employing diffusion weighted MRI and language testing.

**Methods:**

Twenty-three children with RE and 23 matched controls (age: 8–14 years) underwent structural (T1-weighted) and diffusion-weighted MRI (b = 1200 s/mm^2^, 66 gradient directions) at 3T, as well as neuropsychological language testing. Combining tractography and a cortical segmentation derived from the T1-scan, the rolandic tract were reconstructed (pre- and postcentral gyri), and tract fractional anisotropy (FA) values were compared between patients and controls. Aberrant tracts were tested for correlations with language performance.

**Results:**

Several reductions of tract FA were found in patients compared to controls, mostly in the left hemisphere; the most significant effects involved the left inferior frontal (p = 0.005) and supramarginal (p = 0.004) gyrus. In the patient group, lower tract FA values were correlated with lower language performance, among others for the connection between the left postcentral and inferior frontal gyrus (p = 0.043, R = 0.43).

**Conclusion:**

In RE, structural connectivity is reduced for several connections involving the rolandic regions, from which the epileptiform activity originates. Most of these aberrant tracts involve the left (typically language mediating) hemisphere, notably the pars opercularis of the inferior frontal gyrus (Broca’s area) and the supramarginal gyrus (Wernicke’s area). For the former, reduced language performance for lower tract FA was found in the patients. These findings provide a first microstructural white matter correlate for language impairment in RE.

## Introduction

Rolandic epilepsy (RE) is an idiopathic localization-related epilepsy of childhood characterized by centro-temporal spikes on EEG, i.e. originating from the rolandic (sensorimotor) cortex. Seizures are usually mild and nocturnal, and involve hemifacial spasms and speech arrest [1], [2]. The typical age at seizure onset is 7–10 years and spontaneous remission of seizures is observed during adolescence, usually before the age of 16 years [2]. RE is classically considered a benign condition, and is therefore also known as benign epilepsy of childhood with centro-temporal spikes (BECTS).

However, over the last years RE has been associated with visuomotor impairments and problems in spatial perception and orientation, but also with psychiatric disorders, lower IQ, dyscalculia and dyslexia [3]. Not only has the presence and severity of these comorbidities put the assumed benign character of RE under debate [3]; more importantly, their nature hints at dysfunction in neuronal circuits distant from the rolandic focus, rather than purely sensorimotor pathology [4].

Despite the wide range of associated cognitive impairments, especially impairments of the language system have frequently been reported. These vary from verbal memory impairment and auditory deficits to reading disability and speech sound disorder [5], [6], [7], [8]. Functional imaging, combined with neuropsychological testing, suggests that especially anterior language areas are affected [9], [10]. Abnormalities in functional as well as structural connectivity have been found in other types of childhood epilepsy [11]. Moreover, investigations of conventional MRI scans have revealed structural abnormalities in RE [12], including white matter hyper-intensities [13]. Also, patterns of subtle cortical thickness abnormalities have been described [14]. These findings prompt for an investigation into the underlying white matter connections (and the association with language impairment) in RE.

Given the origin of the epileptiform activity in the rolandic regions, we hypothesize that the white matter tracts connecting to these regions may be compromised, and that these abnormalities may be related to the language impairments. We employ diffusion-weighted MRI to reconstruct these tracts (tractography) and to quantify their microstructural integrity in a group of children with RE and a cohort of age-matched controls. Potentially aberrant tracts are interpreted in the light of language impairment based on their topology and possible associations with a neuropsychological measure for language performance. In addition and for completeness, we tested for voxel-wise abnormalities in white matter integrity using a robust and well-established technique (tract based spatial statistics; TBSS).

## Materials and Methods

### Ethics statement

Written informed consent was obtained from all parents or guardians and the study was approved by the review boards of both Maastricht University Medical Center and epilepsy center Kempenhaeghe. The study is registered at www.clinicaltrials.gov under number NCT01335425.

### Subjects

A clinical cohort of 23 children with RE (11.4±2 years; range: 8.0–14.6 years; 9 girls) were recruited at the specialized epilepsy referral centre Kempenhaeghe; for inclusion criteria, see below. An age- and gender-matched healthy control cohort was also included (N = 23; 10.4±1.6 years; range 8.1–14.0 years; 11 girls). For further subject characteristics, see Table 1.

**Table 1:** Subject characteristics, if applicable mean±SD.

Subject characteristics		
	Patients	Controls
N (m/f)	14/9	12/11
Age [y]	10.4±1.6	11.4±2.0
Age at epilepsy onset [y]	7.5±2.1	n.a.
Epilepsy duration [y]	3.9±2.1	n.a.
Handedness (r/l/ambi)	19/3/1	21/2/0
Number of AEDs (0/1/>1)	13/6/4	n.a.

AED stands for anti-epileptic drug; n.a. for not applicable.

### Inclusion criteria

Patient selection was based on seizure semiology and electrophysiological (EEG) data as acquired during the diagnostic work-up. For this, criteria from literature were used [2], [15]. EEG criteria included the presence of spike and slow wave complexes occurring as individual paroxysms or in repetitive clusters with a maximum in the mid temporal and/or central electrodes and with a temporal-frontal dipole field. Additional independent central, mid temporal, parietal or occipital spike wave foci in the same or other hemisphere were allowed. To exclude severe cases (Landau-Kleffner syndrome (LKS) or LKS-like), interictal epileptiform activity was required to be present <85% of the time during non-REM sleep. With respect to seizure semiology, seizures with anarthria, hemiclonia involving the face and/or unilateral extremities, or secondarily generalized seizures were considered. In case of poorly observed nocturnal seizures (3 cases), post-ictal signs of a generalized seizure or confirmation of post-ictal hemiparesis were sufficient for inclusion in case of otherwise typical EEG.

The children with RE were tested using the Wechsler Intelligence Scale for Children, third revised edition, Dutch version (WISC-3), and all had a full-scale IQ >70. None of the healthy controls had (a history of) dyslexia, learning and/or psychiatric disorders, nor attended special education. Children were excluded if they had dental braces (MRI quality) or were somewhat afraid in the scanner.

### Language assessment

All children were subjected to the Clinical Evaluation of Language Fundamentals test, 4^th^ edition (CELF-4), Dutch version [16], [17]. The CELF is a verbally presented language test, which is the gold standard for language assessment in children and adolescents (5–21 years). It was used to assess the core language score, which is a global measure for overall language performance.

### MR imaging

Structural T1-weighted imaging was performed at 3T (Philips Achieva, Best, the Netherlands) using a receive-only SENSE head-coil. A 3D fast-spoiled gradient echo sequence was used employing echo time/repetition time/inversion time (TE/TR/TI) 3.8/8.3/1022 ms at a resolution of 1×1×1 mm^3^. The acquisition time was 8 min.

The T1-weighted scans were reviewed by a board certified neuroradiologist with >20 years of experience (PH). No relevant structural abnormalities were found.

High angular resolution diffusion-weighted imaging (HARDI) was performed using a set of 66 gradient directions distributed evenly over the sphere [18], employing a diffusion-sensitizing b-value of 1200 s/mm^2^. In addition, a single minimally diffusion-weighted image (b0-scan) was acquired. Other settings were: TE/TR 72/6600 ms, resolution 2×2×2 mm^3^, and acquisition time 9 min.

### Cortical parcellation

The T1-weighted structural image was parcellated into 35 cortical regions per hemisphere using the Freesurfer software package as available at http://surfer.nmr.mgh.harvard.edu/. The steps involved are brain extraction, tissue segmentation, and cortical parcellation based on image intensity (gradients) in a template-driven, fully automated and highly robust way [19], [20], [21]. The resulting cortical parcellation of in total 70 regions is gyral pattern based and includes, among others, the left and right pre- and postcental gyrus (henceforth referred to as the 4 rolandic regions).

The cortical parcellation was transformed into diffusion space by affine registration to the b0-scan using FSL routines (FMRIB’s Software Library, Oxford, UK).

### Tractography

**Preprocessing.** Preprocessing and tractography were performed as described previously [22]. Diffusion-weighted sequences were movement-corrected by affine registration to the b0-scan using CATNAP (Coregistration, Adjustment, and Tensor-solving: a Nicely Automated Program, 2008). This included correction of the gradient directions for the corresponding rotations [23], [24].

**Constrained spherical deconvolution.** Voxel-wise fiber orientation distributions (FODs) were estimated using constrained spherical deconvolution (CSD, [25], [26]) as implemented in the MRtrix software package, see http://www.nitrc.org/projects/mrtrix/. As opposed to the conventional diffusion tensor (DT), CSD FODs can represent multiple fiber orientations per voxel and as such account for partial volume effects induced by within-voxel fiber crossing, kissing, bending and fanning [27].

Diffusion tensor imaging (DTI) was employed to calculate fractional anisotropy (FA) maps [28]. For each subject, the response function needed for CSD was estimated from the data by averaging the diffusion profiles of high FA voxels (FA>0.7), aligned along the direction of their principal eigenvector. To ensure that the selected voxels represent (deep) white matter, an eroded brain mask was applied (5 single voxel erosion iterations). For more information on the CSD methodology, we refer to our previous work [22].

CSD FODs are expressed in spherical harmonics, much as (periodic) time signals can be expressed as Fourier series [25]. Higher orders can represent better resolved FODs, with sharper lobes. A spherical harmonic order l_max_ of 8 was used (corresponding to 45 spherical harmonics), which is assumed to adequately resolve crossings without over-fitting the data [27].

**Whole brain tracking and investigated pathways.** For each subject, a whole-brain tractogram was constructed, consisting of 5.000.000 streamlines seeded randomly throughout the brain. Probabilistic CSD tractography was used as implemented in MRtrix. This implies that streamline propagation is allowed in any FOD direction above a certain amplitude threshold, rather than only in the direction of FOD maxima. This allows the streamlines to disperse (to a limited extent) over the width of the FOD lobes, exploring connectivity over a certain angular range. The tractography settings included an FOD amplitude threshold of 0.1, a propagation step size of 0.2 mm, a minimum curvature radius of 1 mm and minimum/maximum streamline lengths of 10/200 mm, respectively.

In an approach similar to Rose et al. [29], for each of the 4 rolandic areas as obtained from the (b0-registered) Freesurfer parcellation, the connectivity to the 69 other cortical regions (both ipsi- and contralateral) was investigated by iteratively selecting those streamlines from the whole-brain tractogram that passed through both the rolandic (seed) and the target region, leading to a total of 270 potential (unique) connections. Since the number of streamlines depends on the size of the seed and/or target region, the number of streamlines of each connection was normalized by its respective number of seed and target voxels [30]. To discard noise-dominated connections, only those for which this ratio exceeded 5 were selected. This corresponds to approximately 10% of the maximum value of this ratio, and led to a limited number of 30 connections being taken forward for further analysis, see below.

**Connectivity metrics.** For each connection, structural connectivity was quantified by tract FA, which was calculated by constructing a map of the number of streamlines passing through each voxel (tract density imaging (TDI, [31]) and employing this to calculate a weighted average. This method gives more weight to FA values in regions where streamline packing is high.

Since FA is age dependent [32], [33], tract FA values were group-wise corrected for age using linear regression. Group differences in (age-corrected) tract FA values were inferred on using permutation testing (N = 100.000). Results were corrected for multiple comparisons employing false discovery rate (FDR) control at q<10% [29]. For the aberrant connections, the (age corrected) tract FA values were correlated with core language scores to test for associations between structural connectivity and language performance.

### Tract-based spatial statistics

To detect possible voxel-wise FA abnormalities, patients were compared to controls employing tract-based spatial statistics (TBSS, [34]) as implemented in FSL. This involves spatial normalization of the individual FA maps and the subsequent construction of a mean (common) FA skeleton. Next, all subjects’ FA data are projected onto this skeleton and voxel-wise cross-subjects statistics is applied to test for group differences (permutation testing, N = 5000). TBSS is assumed to have higher sensitivity than conventional voxel-wise comparison of DT metrics, among others by improved spatial normalization. Since its introduction, TBSS has rapidly become accepted in the field, and has been repeatedly applied to a range of neurological diseases including epilepsy [29], [34], [35], [36], [37], [38].

Results were enhanced for clustered effects using threshold-free cluster enhancement (TFCE, [39]) and corrected for multiple comparisons at p<0.05 within the TBSS tool. In line with the tract-wise approach, the TBSS analysis was also corrected for age using group-wise linear regression.

## Results

### Language performance

The core language score of the patients was 94±17, which is significantly reduced compared to the controls (106±11; p = 0.007, Student’s t-test).

### Rolandic connectivity

**Figure 1:**
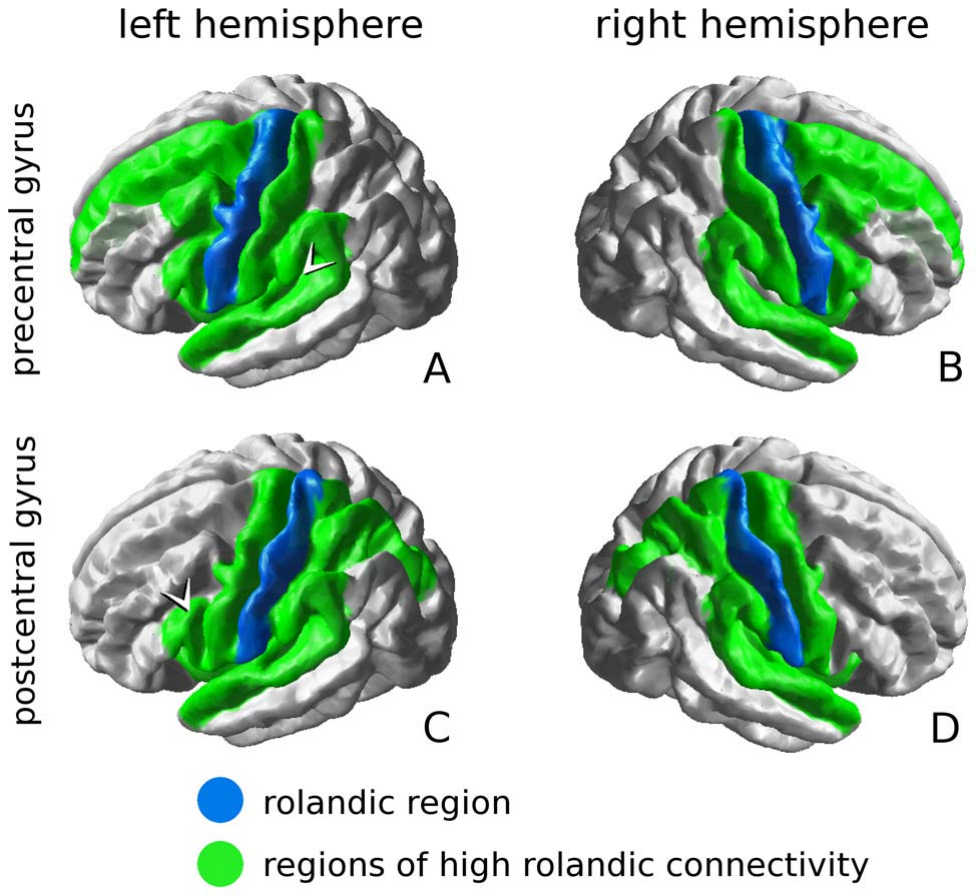
Rolandic connectivity profiles. The 10% most robust rolandic connections, for which the number of streamlines/voxel >5. Averaged over all subjects, all 4 rolandic regions show high perisylvian connectivity, connecting strongly to the supramarginal and superior temporal gyri. Furthermore, the precentral gyri also strongly connect to prefrontal regions (A, B), whereas the postcentral gyri show strong connectivity with the superior parietal cortex. For the precentral gyri, the interhemispheric difference lies in the transverse temporal gyrus (arrowhead in A); for the postcentral gyri, the differences in connectivity lies in the pars opercularis of the inferior frontal gyrus (arrowhead in C).

For each of the 4 rolandic regions, the robust connectivity patterns for the pooled subjects (patients and controls combined) are given in Figure 1. These prominent connections were confined to the ipsilateral hemisphere and had several features in common. In both hemispheres, the pre- and postcentral gyrus showed strong mutual connectivity, and each rolandic region was strongly connected with the superior temporal and supramarginal gyrus (i.e. perisylvian cortex). Also the insula and the paracentral gyrus (adjacent to the rolandic cortex, in the medial plane) were among the regions of prominent connectivity for all 4 rolandic regions.

Specific for the bilateral precentral gyri was high connectivity to several (pre)frontal regions, i.e. the superior and caudal middle frontal cortex, and the pars opercularis of the inferior frontal gyrus (IFG), see Figure 1 A, B. Furthermore, high connectivity was found with the transverse temporal gyrus (adjacent to the insula), but only in the left hemisphere (arrowhead in Figure 1 A).

Furthermore, both postcentral gyri specifically showed strong connectivity with the superior parietal and the transverse temporal gyri, see Figure 1 C, D. Only for the left postcentral gyrus, strong connectivity was found with the pars opercularis of the IFG (arrowhead in Figure 1 C).

### Aberrant tract fractional anisotropy

**Figure 2:**
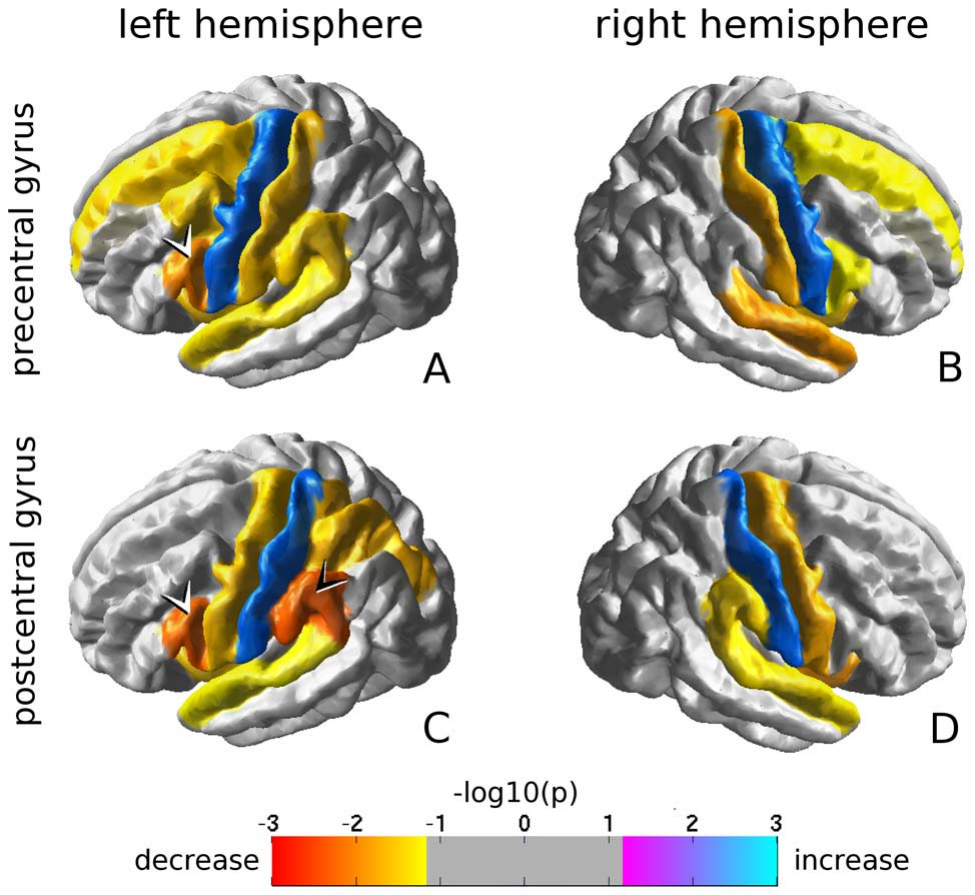
Abnormalities in rolandic connectivit. Only reductions in tract FA were found for patients compared to controls, the most extensive and significant of which were located in the left hemisphere. Especially notice the FA reduction for the rolandic connections with the pars opercularis of the inferior frontal gyrus (white arrowheads in A and C), and the postcentral connection with the supramarginal gyrus (black arrowhead in C).

Several significant reductions in tract FA were found in patients compared to controls, see Figure 2. No connections were found for which FA values were higher in patients than in controls.

General features are reduced tract FA for the connections between the pre- and postcentral gyri in both hemispheres, as well as reduced tract FA for the connections between each of the 4 rolandic regions and the ipsilateral insula and superior temporal gyrus.

For the precentral gyri, additional reductions in tract FA were found for the connections with the superior frontal cortex and the pars opercularis of the IFG. Specific for the left precentral gyrus was a reduction in tract FA for the connection with the caudal middle frontal cortex (p = 0.03), which was not found at the right.

For the postcentral gyri, additional reductions in tract FA were found for the connections with the supramarginal gyri. This effect was more pronounced in the left than in the right hemisphere (p = 0.005 compared to p = 0.03, respectively), see the black arrow in Figure 2 C. Furthermore, for the left precental gyrus, tract FA was reduced for the connection with the pars opercularis of the IFG (p = 0.006), see the white arrow in Figure 2 C. Again, a comparable effect in the right hemisphere was absent.

### Associations with language performance

**Figure 3:**
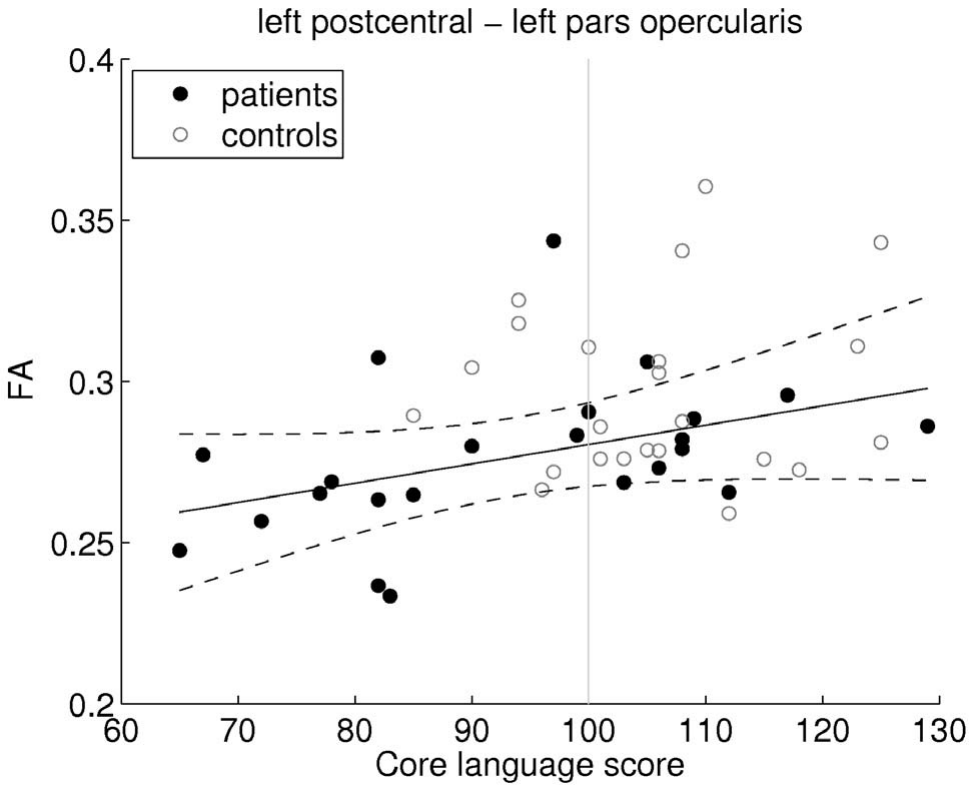
Association between reduced rolandic connectivity and lower language performance. In the children with RE, lower tract FA values were significantly correlated with lower core language scores for the connection between the left postcentral gyrus and the pars opercularis of the left inferior frontal gyrus (p = 0.043, Pearson’s R = 0.43). Regression line (solid) and 95% confidence interval (dashed) in black; norm core language score (100) indicated by vertical gray line. FA values were age-corrected.

For several of the aberrant connections, significant correlations were found between core language score and (age corrected) tract FA. In the patients, lower core language scores were associated with lower tract FA for the connection between the left postcentral gyrus and the pars opercularis of the left IFG (p = 0.043, Pearson’s R = 0.43), see Figure 3. Similar effects were found for three connections involving the right precentral gyrus, i.e. the connection with the right postcentral gyrus (p = 0.030, R = 0.45), the connection with the right paracentral gyrus (p = 0.016, R = 0.50), and the connection with the right superior frontal cortex (p = 0.023, R = 0.47).

No significant correlations were found in the controls.

### Tract-based spatial statistics

TBSS revealed no significant FA differences between patients and controls.

## Discussion

This study was set out to find white matter abnormalities involving the rolandic (sensorimotor) cortex in children with RE. We hypothesized rolandic connections to be disturbed in this type of epilepsy since it is characterized by rolandic epileptic foci. Moreover, since RE is associated with language problems, we explored whether potential connectivity abnormalities might be linked to language impairment.

### Major findings

We identified anatomically plausible patterns of connectivity for the rolandic regions, generally displaying high centro-temporal connectivity, and specific additional frontal and parietal connections for the precentral and the postcentral gyri, respectively. With respect to connectivity abnormalities, our main findings are:

1. Significant reductions in tract FA were found in patients compared to controls, indicative of reduced structural connectivity;
2. More connectivity reductions were found in the left (typically language dominant) hemisphere than in the right;
3. Several of these abnormalities were more pronounced in the left hemisphere (i.e. higher significance) than in the right;
4. For the connection between the left postcentral gyrus and the pars opercularis of the left IFG, lower tract FA was associated with lower core language scores in the patients.

### Patterns of connectivity

The patterns of rolandic connectivity are given in Figure 1. The connectivity patterns are highly symmetric, with the exception of 2 connections that are specific for the left hemisphere, i.e. the connection between the precentral gyrus and the transverse temporal gyrus, and that between the postcentral gyrus and the pars opercularis (arrowheads in Figure 1). Note that generally, the left hemisphere is language dominant; more specifically the left pars opercularis (or rather, the left IFG as a whole) is considered important for expressive language (Broca’s area) [40], [41]. Our findings suggest that specifically the left pars opercularis is structurally integrated with the rolandic regions, which implies it might inherently be affected by rolandic neuropathology, such as epileptiform activity in RE. In line with this, we observed that for all 4 rolandic regions, the superior temporal and supramarginal gyrus of the perisylvian cortex are among the important connections. The perisylvian cortex of the left hemisphere is associated with language, covering Broca’s area in the inferior frontal gyrus, Wernicke’s area in the supramarginal gyrus, and areas relevant for reading in the temporal lobe [42], [43].

### Reductions in tract fractional anisotropy

The investigation of tract FA demonstrated reduced rolandic connectivity in patients compared to controls, predominantly in the left hemisphere. The most significant effects involved tracts connecting the rolandic cortex to the pars opercularis and supramarginal gyrus of the left hemisphere (arrowheads in Figure 2). Since both represent language-mediating regions, the reductions in structural connectivity might be relevant in the context of language impairment.

On the other hand, reduced language lateralization and/or reorganization of language to the right hemisphere have recently been described in RE [44], [45], [46]. This relative increase of right-hemispheric involvement in language may (partly) compensate for the reported abnormalities in structural connectivity of left-lateralized language connections.

### Associations with language performance

We further investigated the relevance of the aberrant connections for language impairment by correlating their connection strength with language performance.

Significant effects were found for the patients only, in which lower tract FA values were associated with lower core language scores. Among others, this was the case for 2 central connections, i.e. those between the right precentral gyrus and the right postcentral and paracentral gyri, respectively. Because of the proximity of these connections to the epileptic focus, we assume these associations merely imply that more severe primary (rolandic) pathology is (indirectly) related to more pronounced cognitive impairment, among others lower language performance. Similarly, the association between lower tract FA values and lower core language scores for the connection between the right precentral gyrus and the right superior frontal cortex may also just provide an indirect window on general cognitive impairment, given the importance of the frontal lobe in cognitive processing. In fact, reduced connectivity of the frontal lobe has been related to reduced cognitive performance in childhood epilepsy before [11].

On the other hand, the association between lower tract FA values and lower core language scores for the connection between the left postcentral gyrus and the pars opercularis of the left IFG is expected to be specifically related to language impairment, since this connection provides a link between a rolandic region and a language area (Broca’s). Note that in language mediating connections such as this, a positive correlation between FA and language performance is also expected in the controls, as increased structural integrity may lead to improved cognitive performance. Possibly this lack of association in the controls is due to the narrower range of core language scores. This suggestion was also made concerning comparable findings with respect to functional connectivity in patients with cryptogenic localization-related epilepsy by our group [47].

### Integration of the motor and language system

The question why loss of structural connectivity *between* the rolandic (sensorimotor) regions and the language system (left pars opercularis and supramarginal gyrus) might impact functionality *within* the language system needs to be addressed. The relevance of Broca’s area for expressive language has long been established, and might hinge on the relevance of this region as (pre)motor area relevant for the coordination of complex articulatory movement. However, several recent studies have demonstrated that the motor and the language system are more fundamentally integrated [48], [49], [50], [51], [52]. For example, it has been demonstrated that speech sounds predominantly activate the left supramarginal gyrus (Wernicke’s area) but, depending on the articulator involved (lips or tongue), differently activate the associated regions in the motor cortex [48]. This implies language functionality within the motor cortex, in other words: the motor and the language system have an (at least partly) shared neuronal substrate, and in that sense cannot be viewed as separate entities [48]. For an overview, see Cappa et al. [53], and the references therein. The rolandic connectivity patterns we identified are in line with this view as they demonstrate strong connectivity between the (in the left hemisphere language mediating) supramarginal cortex and the rolandic regions. From this, it can be speculated that rolandic neuropathology might inherently influence the language system, as is consistent with our findings of reduced structural integrity of these connections. Furthermore, if the role of Broca’s area in the language network is not purely expressive, impairments within this region might cause a range of language problems. A number of studies exist on the role of Broca’s area beyond expressive language [54], [55], for an overview we refer to Burns et al. [56]. These findings fits to the broad profile of language impairment as found in RE, including reading disability, speech sound disorder, verbal memory impairment, neurophysiologic auditory deficit and abnormalities in oromotor and dichotic listening performance [5], [6], [7], [8], [57].

All this is in line with our finding of reduced tract FA (and core language score) in the connection between the left postcentral gyrus and the pars opercularis of the left IFG.

### No voxel-wise FA abnormalities

TBSS revealed no abnormalities, which might imply higher sensitivity of analysis on the tract level rather than voxel-wise inference, at least in the case of a clearly localized hypothesis, such as our case of aberrant rolandic connectivity in RE. This might (partly) be related to group-wise overlap of abnormalities on the tract level, but not on the (more local) voxel level. Note that TBSS projects subject-specific FA values onto a common (group-specific) FA skeleton, already improving spatial regularization with respect to conventional (whole-brain) voxel-wise techniques.

### Methodological considerations and outlook

In this study, we investigated connectivity between relatively large cortical regions as defined by an automated, gyral pattern based cortical parcellation (Freesurfer). These regions might not have been optimal to fully and unambiguously segment the underlying major white matter tracts, but allowed us to investigate the connectivity of specific gyral features of interest, i.e. the pre- and postcentral gyri (rolandic regions). The Freesurfer parcellation has been shown to be robust (Desikan et al., 2006), and ensured objectivity and consistency over subjects. Furthermore, it has been successfully applied for investigations of structural connectivity before [29], [58].

Our aim was to examine aberrant rolandic connectivity in RE. Although our interest was in language impairment, this methodology was not biased towards finding aberrant connectivity with classical language mediating areas. Nonetheless, the most pronounced impairments in structural connectivity were found in the left hemisphere, more specifically in the left supramarginal gyrus (Wernicke’s area) and the pars opercularis of the left IFG (Broca’s area). What is more, for the latter it was demonstrated that lower tract FA values were associated with lower language performance. These findings are in line with the view that language impairment is a prominent aspect of the cognitive problems seen in RE. For future research, it would be interesting to study structural connectivity abnormalities in RE in classical language-mediating white matter bundles such as the arcuate fasciculus [41], [59]. However, such analyses required dedicated approaches [60], and are beyond the methodological scope of this study.

In this work, we quantified structural connectivity using FA. FA is at best an indirect measure for white matter integrity, and its accuracy is compromised at regions of fiber crossing, kissing, bending and fanning [61], [62]. However, in our experience FA has good reproducibility and subject-differentiating power on the tract level [22]. Indeed, at present FA is still the measure of choice in most investigations of structural connectivity [29], whereas other measures for white matter integrity may also be used, such as apparent diffusion coefficient (ADC), number of streamlines per seed/target voxel, tract volume, or the recently introduced apparent fiber density (AFD; [63]). Each of these measures has a different interpretation and they also vary in reproducibility (and therefore in sensitivity for abnormalities) [22]. In our view, the field of tract connectivity measures other than FA is underexplored and forms an interesting subject for future research. The use of measures not derived from the diffusion-weighted data itself, such as magnetization transfer ratio (MTR) imaging to obtain estimates for the degree of myelination [58], even further broadens the scope of possibilities.

Finally, our results were robust with respect to FDR correction for multiple comparisons at q = 10%. In a methodologically similar approach, the same q-value was applied [29]. For future studies it remains to be investigated whether our results are robust with respect to more stringent statistical correction employing larger numbers of subjects.

In conclusion, in RE abnormalities in structural connectivity can be found for specific white matter tracts involving the rolandic regions, where the epileptic focus is located. Most of these aberrant tracts involve the left hemisphere, notably the pars opercularis of the inferior frontal gyrus (Broca’s area) and the supramarginal gyrus (Wernicke’s area). For the former, lower structural connectivity was significantly correlated with reduced language performance in the patients. The fundamental integration of the motor and the language system as described in recent literature might explain why impairment of these moto-lingual connections impacts language performance, and might be a key concept in understanding language impairment in RE.
